# Locally Advanced Primary Cervical Endometrial Stromal Sarcoma: A Case Report

**DOI:** 10.7759/cureus.116888

**Published:** 2026-09-25

**Authors:** Asmae Bali, Kaouthar Khater, Fadoua Jebrouni, Hind Chibani, Ouissam Al Jarroudi, Sami Aziz Brahmi, Said Afqir

**Affiliations:** 1 Medical Oncology, Mohammed VI University Hospital, Faculty of Medicine and Pharmacy of Oujda, Mohammed First University of Oujda, Oujda, MAR

**Keywords:** cervical stromal sarcoma, chemotherapy, figo stage iva, immunohistochemistry, molecular alterations

## Abstract

Primary cervical endometrial stromal sarcoma (ESS) is a challenging mesenchymal neoplasm that may present with non-specific clinical manifestations and advanced local disease at diagnosis. We report the case of a 50-year-old diabetic woman presenting with metrorrhagia and pelvic pain. Clinical examination revealed a large ulcerative cervical mass infiltrating the vaginal walls. Histopathology with immunohistochemistry supported the diagnosis of ESS. Imaging demonstrated extensive loco-regional invasion of the bladder, lower vagina, and rectal wall, consistent with FIGO stage IVA disease. The patient was treated with systemic chemotherapy (carboplatin-paclitaxel). This case highlights the diagnostic challenges of cervical ESS, the importance of histopathological and immunohistochemical evaluation, and the therapeutic considerations in locally advanced disease.

## Introduction

Endometrial stromal sarcoma (ESS) is a rare mesenchymal malignancy of the female genital tract arising from endometrial stromal cells. It accounts for a small proportion of uterine malignancies and represents a minority of uterine sarcomas [1,2]. ESS most commonly arises in the uterine corpus, whereas primary cervical involvement is exceptionally rare [2,3]. Because of its unusual anatomical location and non-specific clinical presentation, primary cervical ESS may pose significant diagnostic and therapeutic challenges.

According to the World Health Organization classification of female genital tumors, ESS includes low-grade ESS (LG-ESS) and high-grade ESS (HG-ESS), which differ in their morphological, molecular, and clinical characteristics [1,4]. LG-ESS generally follows a relatively indolent clinical course but may recur after prolonged intervals, whereas HG-ESS is associated with more aggressive behavior and poorer outcomes [1,4]. Molecular alterations have substantially contributed to the classification of these tumors; recurrent fusions involving *JAZF1* and *PHF1* have been described in LG-ESS, while alterations involving *YWHAE-NUTM2* and *BCOR* have been reported in HG-ESS [4-6].

The diagnosis of ESS can be particularly challenging when the tumor arises in an unusual cervical location and is evaluated on limited biopsy specimens because of morphological overlap with other malignant mesenchymal tumors, including leiomyosarcoma and undifferentiated uterine sarcoma [1,2,4]. Immunohistochemical analysis may support the diagnosis and help distinguish ESS from epithelial and smooth muscle tumors; however, no single immunohistochemical marker is sufficiently specific to establish the diagnosis on its own [4,7]. Markers such as CD10, estrogen receptor (ER), progesterone receptor (PR), desmin, and smooth muscle actin (SMA) may provide complementary diagnostic information, while Ki-67 may contribute to the assessment of proliferative activity [4,7]. Molecular testing can further refine tumor classification when available, particularly in diagnostically challenging cases [4-6].

Primary cervical ESS is exceptionally rare, and data regarding its pathological characterization and management remain limited, particularly when the disease presents at an advanced stage with involvement of adjacent organs. Reporting such cases is relevant to illustrating the diagnostic limitations that may arise when comprehensive pathological and molecular characterization is not feasible and to describing the multidisciplinary therapeutic approach used for locally advanced disease. We report a case of locally advanced primary cervical ESS with bladder involvement to contribute to the limited clinical literature on this rare presentation.

## Case presentation

A 50-year-old married woman, gravida 2 para 2, with a history of type 2 diabetes mellitus treated with oral antidiabetic agents, presented with a six-month history of abnormal uterine bleeding, foul-smelling vaginal discharge, and pelvic pain. Her general condition was relatively preserved, with an Eastern Cooperative Oncology Group performance status of 1 [8].

Gynecological examination revealed a large ulcerative and exophytic cervical mass causing complete destruction of the cervix and extending to the vaginal walls. Based on the presenting symptoms and clinical findings, the main differential diagnoses included locally advanced cervical carcinoma, cervical leiomyosarcoma, and other mesenchymal tumors of the cervix.

A cervical biopsy was performed. Histopathological examination revealed a malignant mesenchymal proliferation with extensive tumor necrosis. Microscopically, the tumor showed areas of whorled proliferation composed of relatively uniform stromal cells with oval nuclei, fine chromatin, and relatively smooth nuclear contours (Figure 1). Other areas demonstrated a diffuse proliferation of spindle cells arranged in interlacing fascicles, with mild to moderate nuclear atypia and scant cytoplasm (Figure 2). Immunohistochemical analysis demonstrated diffuse expression of CD10 and vimentin, whereas epithelial markers were negative. In the context of the morphological findings, this immunophenotypic profile supported endometrial stromal differentiation.

**Figure 1:**
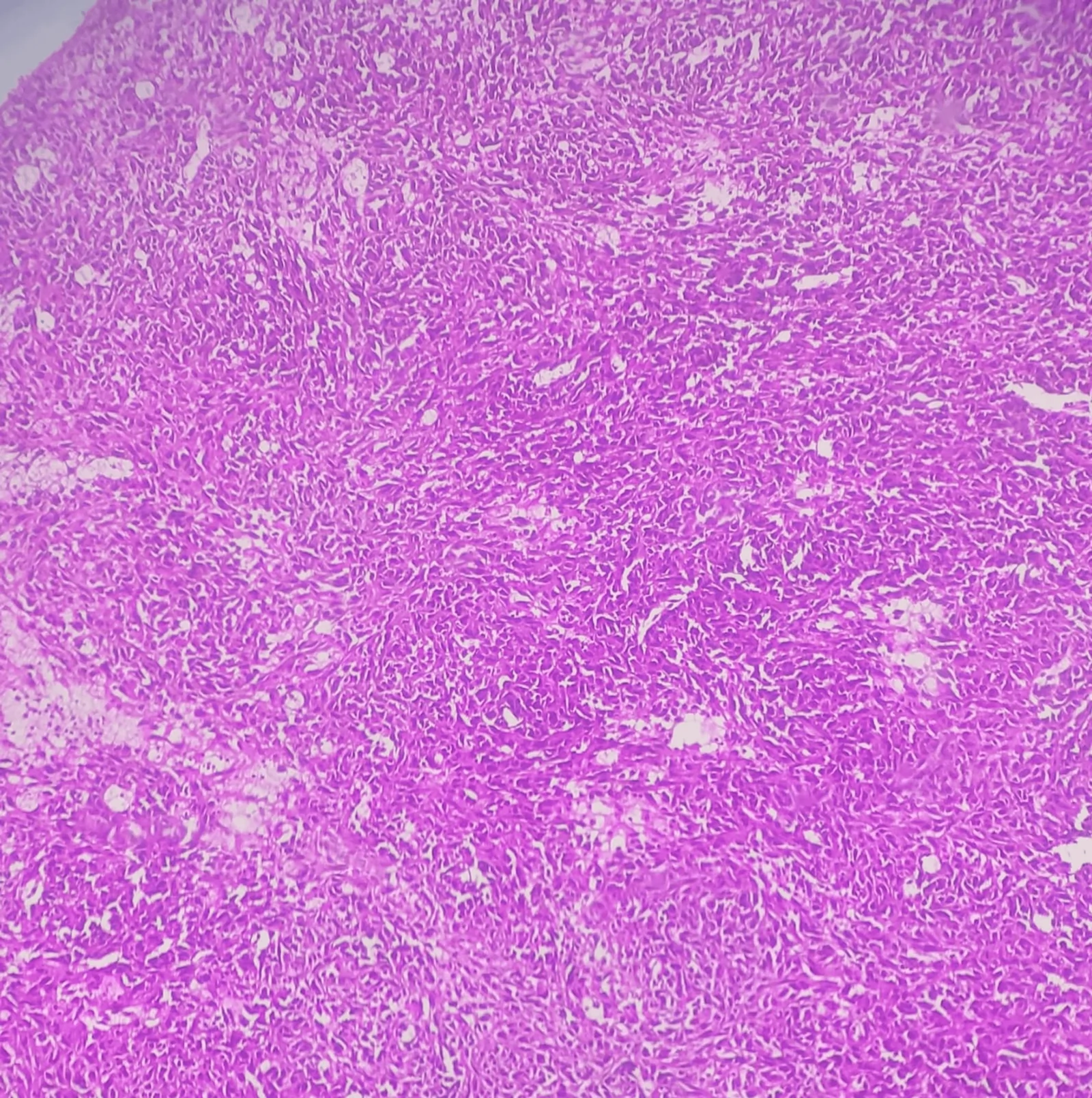
Histopathological features of the cervical tumor. Hematoxylin and eosin (H&E) staining showing a whorled proliferation of relatively uniform stromal cells with oval nuclei, fine chromatin, and relatively smooth nuclear contours (H&E, ×400).

**Figure 2:**
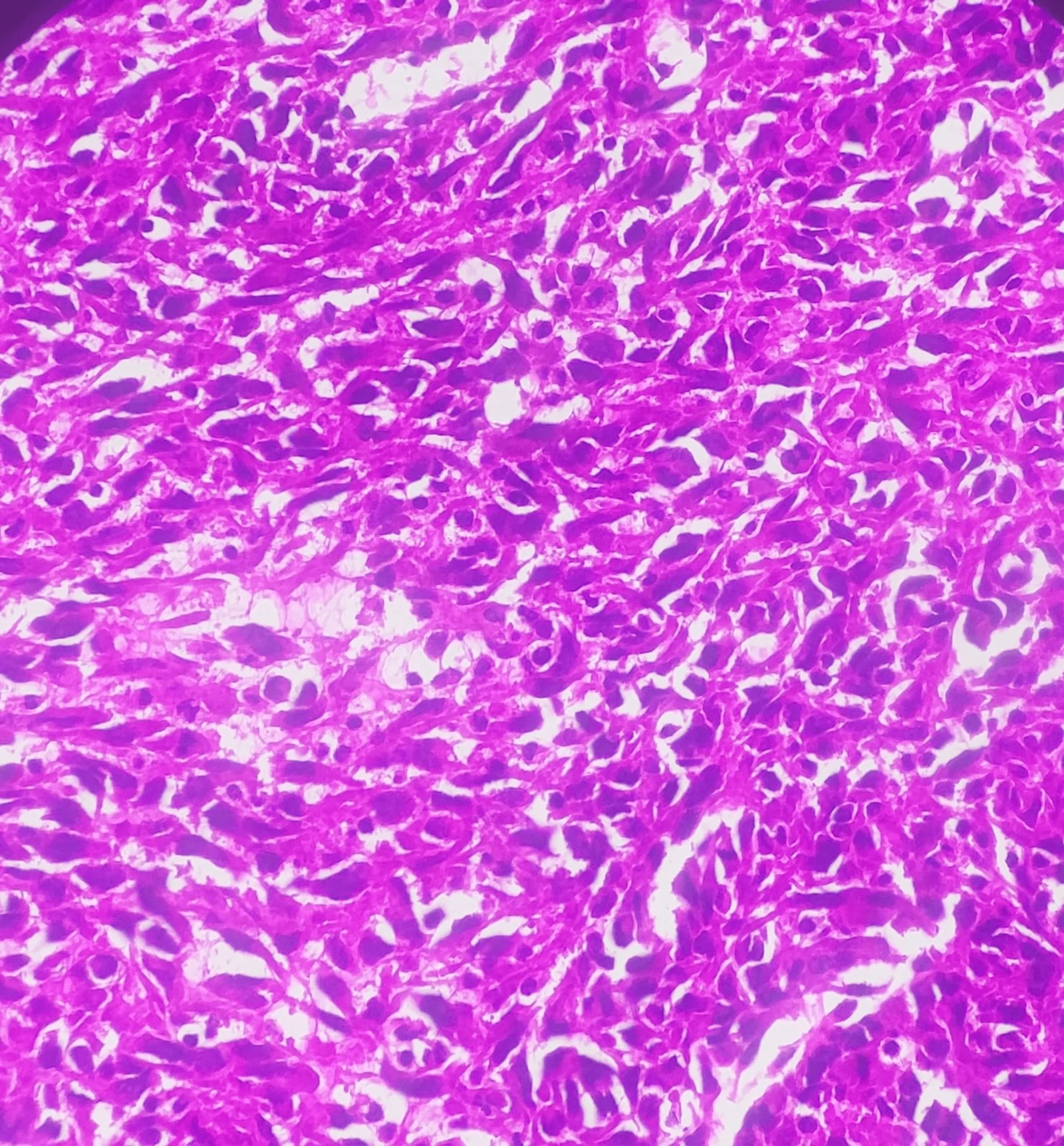
Histopathological features of the cervical tumor. Hematoxylin and eosin (H&E) staining showing a diffuse proliferation of spindle cells arranged in interlacing fascicles, with mild to moderate nuclear atypia and scant cytoplasm (H&E, ×200).

The immunohistochemical assessment was incomplete. ER, PR, desmin, SMA, and Ki-67 were not assessed. Cyclin D1 and *BCOR* expression were also not evaluated. Therefore, the available immunophenotypic findings did not allow definitive histological subtyping or reliable distinction between low-grade and high-grade endometrial stromal sarcoma. Molecular characterization was not performed because molecular testing was unavailable in our clinical setting.

Contrast-enhanced thoraco-abdominopelvic computed tomography (CT) revealed a large cervico-isthmic mass extending to the uterine body, lower third of the vagina, perineum, and urinary bladder, with suspected early involvement of the rectal wall and a right internal obturator lymph node. No distant metastatic lesions were identified. Pelvic magnetic resonance imaging (MRI) confirmed a large cervical mass occupying most of the pelvis and infiltrating the vesical trigone, consistent with locally advanced disease (Figure 3).

**Figure 3:**
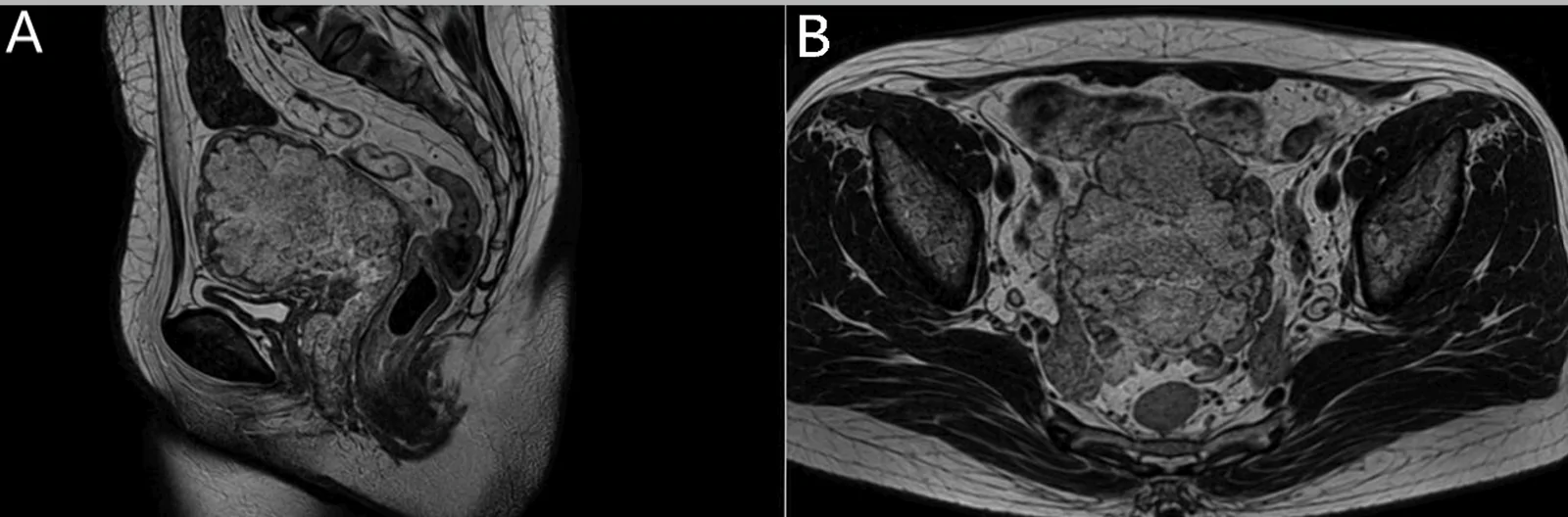
Pelvic magnetic resonance imaging showing a locally advanced cervical mass with infiltration of the vesical trigone. (A) Axial T2-weighted image and (B) sagittal T2-weighted image

Given the locally advanced and unresectable nature of the disease, the case was discussed at a multidisciplinary tumor board. Systemic chemotherapy with carboplatin (area under the curve: 5) and paclitaxel (175 mg/m²), administered intravenously every three weeks, was initiated. The patient received four cycles of chemotherapy. Follow-up CT and MRI demonstrated a partial radiological response, accompanied by marked clinical improvement.

Treatment was generally well tolerated. The main treatment-related adverse event was grade 2 hand-foot syndrome. No other major treatment-related complications were reported.

No definitive surgery was performed. Following systemic chemotherapy and the observed partial radiological response, the patient was referred to the radiation oncology department for evaluation and consideration of locoregional radiotherapy. Radiotherapy was ultimately not administered.

At the last follow-up, performed one month after completion of chemotherapy, the patient remained clinically improved, with no new treatment-related complications reported.

## Discussion

Uterine sarcomas are rare malignant tumors, accounting for a small proportion of uterine malignancies, with ESS representing a minority of these neoplasms [3]. According to the World Health Organization 2020 Classification of Female Genital Tumours, ESS is categorized into LG-ESS and HG-ESS entities based on morphological and molecular characteristics [2]. Primary cervical localization is exceptionally uncommon and may pose significant diagnostic difficulty because it can clinically mimic more prevalent cervical carcinomas. In the present case, extensive urinary bladder invasion indicated a locally advanced cervical presentation corresponding to FIGO stage IVA when assessed according to cervical carcinoma staging criteria [9,10].

Histologically, LG-ESS is composed of small, relatively uniform cells resembling proliferative-phase endometrial stromal cells, with minimal atypia and generally low mitotic activity. In contrast, HG-ESS demonstrates marked nuclear atypia, increased mitotic activity, necrosis, and, in some molecular subtypes, a prominent round-cell component [2,4]. Given the morphological overlap with leiomyosarcoma, undifferentiated uterine sarcoma, and poorly differentiated carcinoma, immunohistochemistry is an important component of the diagnostic work-up. ESS commonly expresses CD10 and may express ER and PR, whereas epithelial markers are generally absent [4,9]. However, these immunohistochemical findings are not entirely specific and should be interpreted in conjunction with morphological findings and, when available, molecular data.

In our patient, the tumor showed diffuse expression of CD10 and vimentin, with negativity for epithelial markers, supporting a mesenchymal tumor with an endometrial stromal phenotype. However, the immunohistochemical characterization was incomplete. Additional markers, including desmin, SMA, ER, PR, and Ki-67, were not performed. Cyclin D1 and *BCOR* expression were also not assessed. Therefore, although the available pathological findings supported an endometrial stromal phenotype, they were insufficient to establish with certainty the specific molecular subtype or to definitively distinguish LG-ESS from HG-ESS. This limitation is particularly relevant because these entities differ in their biological behavior and may have different therapeutic considerations.

Prognosis is influenced by histologic subtype and stage at diagnosis. Population-based analyses have demonstrated differences in long-term survival between LG-ESS and HG-ESS, while advanced-stage disease remains an important adverse prognostic factor [11]. LG-ESS is generally characterized by a relatively indolent clinical course but may recur after prolonged disease-free intervals, whereas HG-ESS typically exhibits more aggressive behavior, with a higher risk of early metastatic dissemination and poorer overall survival [3,11]. In the present case, the locally advanced cervical presentation with urinary bladder invasion represented an unfavorable clinical setting, although the absence of complete pathological and molecular characterization precluded definitive assignment to either LG-ESS or HG-ESS.

From a molecular standpoint, genetic alterations are central to the current classification and pathogenesis of ESS. LG-ESS is strongly associated with recurrent chromosomal rearrangements, most notably the *JAZF1-SUZ12* fusion resulting from t(7;17)(p15;q21), as well as other fusions involving *PHF1*, including *EPC1-PHF1* and *MEAF6-PHF1* [4]. These alterations implicate dysregulation of chromatin remodeling and epigenetic mechanisms in tumorigenesis. In contrast, HG-ESS may harbor the *YWHAE-NUTM2* fusion resulting from t(10;17)(q22;p13), which is associated with high-grade morphology and aggressive clinical behavior [8]. Alterations involving *BCOR*, including *BCOR* internal tandem duplication and *ZC3H7B-BCOR* fusion, have also been described in high-grade ESS and are associated with distinctive morphological and biological features [7].

Importantly, none of these molecular alterations was demonstrated in our patient because molecular profiling was not performed. These molecular abnormalities are presented solely to provide the current diagnostic and biological context of ESS and should not be interpreted as molecular findings specific to this case. Molecular testing could have provided additional diagnostic information, particularly for distinguishing between different ESS subtypes, but its absence represents an important limitation of the present case.

Therapeutically, total hysterectomy with bilateral salpingo-oophorectomy remains the standard surgical approach for localized and resectable ESS [3,5]. Because many LG-ESS tumors express ER and PR, hormonal therapy with progestins or aromatase inhibitors represents an important systemic treatment option in advanced, recurrent, or metastatic disease, particularly in hormone receptor-positive tumors [3]. In our patient, ER and PR status was not assessed; therefore, the potential role of endocrine therapy could not be adequately evaluated.

For unresectable locally advanced or metastatic disease, systemic chemotherapy may be considered. Doxorubicin-based regimens remain commonly used in advanced uterine sarcomas, while platinum-taxane combinations, including carboplatin-paclitaxel, are also used in clinical practice, although prospective evidence specifically addressing ESS remains limited [3,5]. In our case, because of the extensive local invasion and the absence of a feasible curative surgical approach, the case was discussed in a multidisciplinary tumor board, and systemic chemotherapy with carboplatin-paclitaxel was selected. The patient received four cycles, with a partial radiological response and marked clinical improvement.

Radiotherapy may be considered in selected patients to improve local control, particularly in cases with extensive locoregional disease, although its impact on overall survival remains uncertain [3]. In our patient, following systemic chemotherapy and the observed partial radiological response, referral to the radiation oncology department was made for evaluation and consideration of locoregional radiotherapy. However, radiotherapy was ultimately not administered. This therapeutic decision should therefore not be interpreted as evidence against the potential role of radiotherapy in selected patients with locally advanced ESS.

This case has several limitations. First, the pathological characterization was incomplete, with a limited immunohistochemical panel and no assessment of ER, PR, desmin, SMA, Ki-67, cyclin D1, or *BCOR*. Second, no molecular profiling was performed, preventing confirmation of a specific molecular subtype such as *JAZF1-SUZ12*-, *YWHAE-NUTM2*-, or *BCOR*-altered ESS. Third, because of these limitations, a definitive distinction between LG-ESS and HG-ESS could not be established. Accordingly, the molecular abnormalities discussed in this report represent established findings from the literature rather than molecular characteristics demonstrated in our patient.

In the context of a locally advanced cervical tumor with urinary bladder invasion corresponding to FIGO stage IVA when assessed according to cervical carcinoma staging criteria, this case illustrates the diagnostic and therapeutic challenges associated with this exceptionally rare presentation. Although the available morphological and immunohistochemical findings supported an endometrial stromal phenotype, comprehensive pathological and molecular characterization would have provided greater diagnostic precision and potentially relevant therapeutic information. Nevertheless, this case emphasizes the importance of multidisciplinary management and of integrating morphological, immunohistochemical, hormonal receptor, and molecular data whenever available. Molecular characterization may provide additional diagnostic and therapeutic opportunities in selected patients, particularly through approaches targeting pathways involved in the molecular classification of ESS.

## Conclusions

Primary cervical ESS is an exceptionally rare malignancy that poses considerable diagnostic and therapeutic challenges. In this case, the morphological and available immunohistochemical findings supported an endometrial stromal phenotype, while the incomplete immunohistochemical assessment and the absence of molecular profiling limited definitive histological and molecular subclassification. The locally advanced presentation with urinary bladder involvement required individualized multidisciplinary management and was associated with a partial radiological response to systemic chemotherapy. This case emphasizes the importance of integrating morphological, immunohistochemical, and molecular data whenever available, particularly in unusual cervical presentations of ESS.
